# Supplementary material for: A Global Screen for Assembly State Changes of the Mitotic Proteome by SEC-SWATH-MS
Source: Cell Syst. 2020 Feb 26;10(2):133–155.e6. doi: 10.1016/j.cels.2020.01.001 (PMC7042714; doi:10.1016/j.cels.2020.01.001)

Q15477 | SKIV2_HUMAN | SKIV2L DDX13 SKI2W SKIV2 W
Monomer MW [kDa]: 137.755 Monomer expected elution fraction: 37

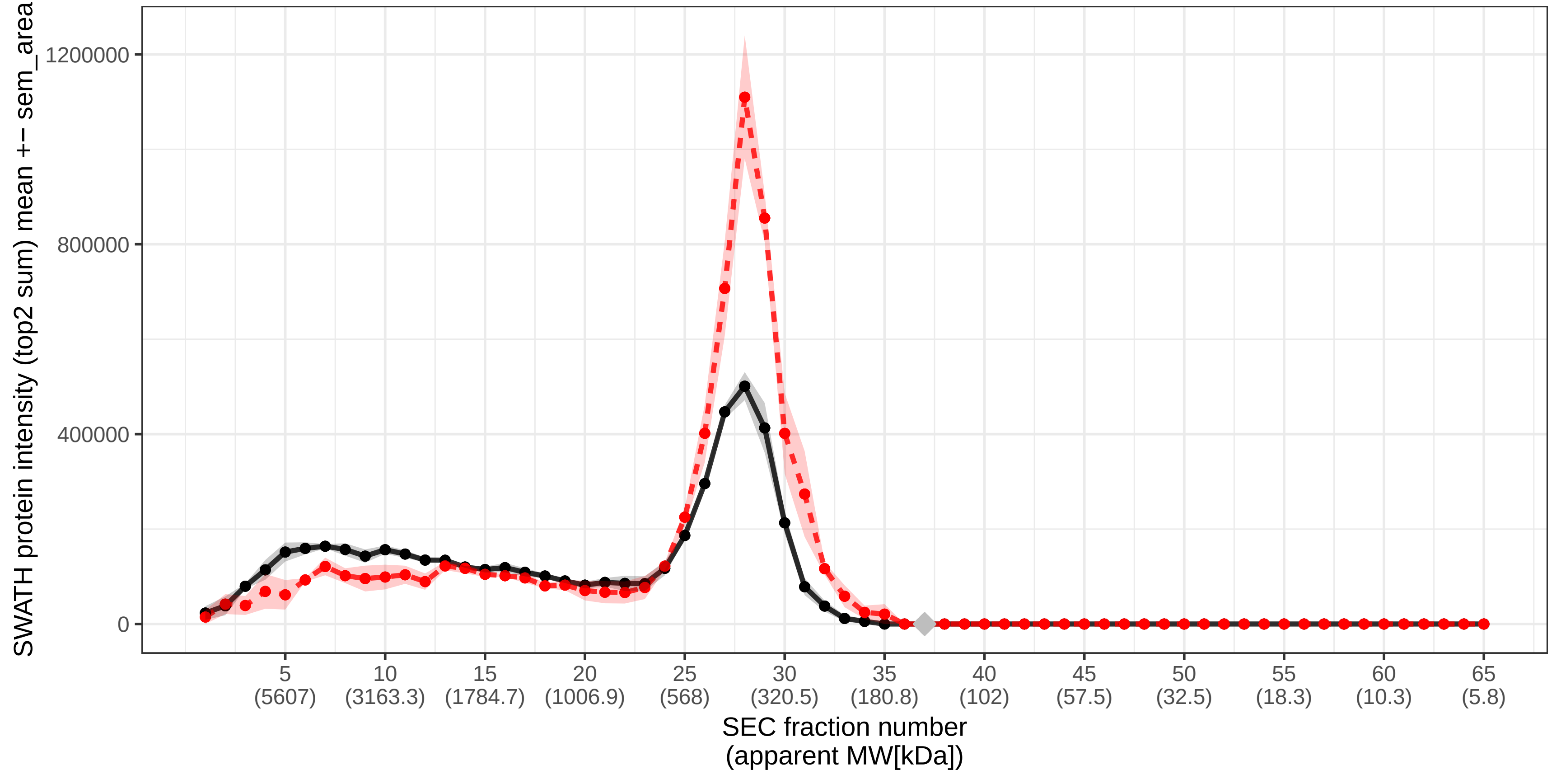

Supplement: Data S1. SEC-SWATH-MS Protein Chromatograms, Related to Figure 1 [file mmc6.zip › SECchrom_Q15477_SKIV2_HUMAN_SKIV2L_DDX13_SKI2W_SKIV2_W.pdf]
